# Flavonoids for MASLD: Hepatic Lipid Targets, Biopharmaceutic Barriers, and Formulation Strategies

**DOI:** 10.3390/ph19091393

**Published:** 2026-09-02

**Authors:** Shuo Yan, Hui Yang, Yingrui Wang, Lejian Zhu, Binsheng Wang, Leiming Zhang, Qing Hao

**Affiliations:** School of Traditional Chinese Medicine, Shandong Medical and Pharmaceutical University, Yantai 264003, China; 18663869393@163.com (S.Y.); yangh2527@163.com (H.Y.); wangyingrui_2002@163.com (Y.W.); zhulejian2000@163.com (L.Z.); wang617424@163.com (B.W.)

**Keywords:** MASLD, MASH, flavonoids, hepatic lipid metabolism, bioavailability, pharmacokinetics, nanodelivery, liver targeting, AMPK, formulation

## Abstract

Metabolic dysfunction-associated steatotic liver disease (MASLD) develops when hepatic lipid acquisition and synthesis exceed the capacity for oxidation and very-low-density lipoprotein export. Flavonoids act on several components of this network, yet their therapeutic development is constrained by poor aqueous solubility, extensive intestinal and first-pass metabolism, variable activity of circulating metabolites, and limited information on hepatic exposure. Experimental studies link representative flavonoids to AMPK–SREBP-1c and PPARα signaling, mitochondrial quality control, Nrf2-dependent redox defense, inflammatory pathways, and the gut–liver axis. By contrast, the available randomized trials of quercetin, hesperidin, anthocyanins, green-tea catechins, EGCG, and soy isoflavones show at most modest changes in liver fat or biochemical markers and do not demonstrate metabolic dysfunction-associated steatohepatitis (MASH) resolution or fibrosis regression. Liposomal, lipid-based, polymeric, and nanocrystal formulations have improved dissolution, systemic exposure, or liver distribution in preclinical models, but comparative pharmacokinetics, chronic safety, manufacturability, and clinical efficacy remain poorly defined. The evidence therefore supports viewing flavonoids as formulation-dependent investigational candidates rather than established MASLD therapies. Progress will depend on chemically standardized products, exposure–response studies, clinically relevant models, and adequately powered trials using validated imaging or histological endpoints.

## 1. Introduction

Metabolic dysfunction-associated steatotic liver disease (MASLD) denotes steatotic liver disease occurring in the setting of cardiometabolic risk [1]. The disorder is common and clinically heterogeneous, ranging from isolated steatosis to metabolic dysfunction-associated steatohepatitis (MASH), fibrosis, cirrhosis, and hepatocellular carcinoma [2,3,4,5,6,7,8]. Liver-related complications are only part of this burden; cardiovascular and other metabolic disorders account for substantial morbidity and must be considered when therapeutic benefit is assessed.

Replacing non-alcoholic fatty liver disease (NAFLD) with MASLD is not simply a change in terminology. The new definition uses positive cardiometabolic criteria while retaining the need to evaluate alcohol exposure and alternative causes of steatosis [1]. At the biological level, insulin resistance, adipose-tissue dysfunction, dietary substrate excess, and de novo lipogenesis interact with mitochondrial, endoplasmic-reticulum, and inflammatory stress. These processes do not contribute equally in every patient, and their relative importance shifts as disease advances; a single target is therefore unlikely to be effective across the full MASLD spectrum [5,9,10,11].

Hepatic fat accumulates when the entry and synthesis of fatty acids outpace their oxidation or export. The liver receives non-esterified fatty acids from adipose tissue and dietary lipids, and it also produces new fatty acids through de novo lipogenesis. Mitochondrial and peroxisomal oxidation, together with very-low-density lipoprotein (VLDL) secretion, counter these inputs [12,13,14,15,16]. Packaging fatty acids as triglycerides can be protective at first. With continued overload, however, diacylglycerols, ceramides, lysophospholipids, and free cholesterol accumulate and disturb insulin signaling while amplifying cellular stress [14,17,18].

Although lifestyle intervention remains first-line care for MASLD, the therapeutic context has shifted. Phase 3 data show that resmetirom can meet endpoints for MASH resolution and fibrosis improvement [19]; in the interim ESSENCE analysis, semaglutide improved both primary histological endpoints at 72 weeks [20]. Against this clinical benchmark, changes in liver enzymes or isolated metabolic markers provide insufficient support for a flavonoid product. Such a candidate would need consistent exposure, demonstrable target engagement, and a hepatic benefit of clinical relevance. Efimosfermin and other metabolic or fibro-inflammatory agents are also in development [21].

Flavonoids are not a uniform chemical class but a broad family of dietary and medicinal polyphenols. In experimental models of fatty liver, reported effects include regulation of metabolism, redox balance, inflammation, autophagy, and gut-derived signals, rather than radical scavenging alone [22,23,24,25,26]. Translation is complicated because many cell experiments use the parent aglycone, whereas the liver in vivo is exposed mainly to glucuronidated, sulfated, methylated, or microbially generated metabolites. Mechanistic interpretation therefore requires attention to the chemical species that actually reach the liver.

Against this background, the objective of this review is to determine whether the mechanistic promise of flavonoids can be reconciled with the doses, chemical species, hepatic exposure, and outcomes examined in humans. We integrate four evidence domains: the lipid biology of MASLD, compound-specific molecular actions, human intervention studies, and formulation approaches designed to improve exposure. Our distinctive perspective is to treat formulation and metabolite-resolved pharmacokinetics as part of the efficacy question rather than as downstream technical considerations. Accordingly, we compare mechanistic findings with pharmacokinetic and clinical observations, distinguish surrogate biomarker changes from disease modification, and identify the evidence needed for a pharmaceutics-led MASLD development strategy.

## 2. Review Methodology

PubMed was searched up to 23 July 2026 for English-language publications, with the main search period extending from January 2000 onward. January 2000 was selected as a pragmatic scope boundary for this structured narrative review to focus the synthesis on contemporary NAFLD/MASLD molecular mechanisms, flavonoid pharmacokinetics, and advanced delivery systems investigated with modern analytical methods; it was not intended to represent a scientifically absolute historical breakpoint. Disease terms (MASLD, MASH, non-alcoholic fatty liver disease [NAFLD], or NASH) were combined with flavonoid-related terms (flavonoid, quercetin, kaempferol, naringenin, naringin, hesperidin, anthocyanin, epigallocatechin gallate, isoflavone, dihydromyricetin, luteolin, apigenin, or fisetin) and with mechanistic or pharmaceutic terms, including lipid metabolism, AMPK, SREBP, PPAR, bioavailability, pharmacokinetics, formulation, nanoparticle, liposome, nanostructured lipid carrier, and liver targeting. A relevant *Pharmaceutics* review was also included to inform the synthesis of formulation studies [27].

Randomized human studies, mechanistic experiments using pathway perturbation, and formulation studies with physicochemical, release, permeability, pharmacokinetic, tissue-distribution, or free-compound comparator data were prioritized. Publications before January 2000 that had already been identified and were directly relevant to foundational concepts were not excluded solely on the basis of date; however, no systematic pre-2000 search or formal backward-citation procedure was performed. Evidence was considered separately as in vitro, animal, observational human, or interventional human evidence. Because the work is a structured narrative review rather than a prospectively registered systematic review, we did not perform a formal risk-of-bias assessment or meta-analysis; the conclusions should be interpreted in that context. 

**Figure 1:**
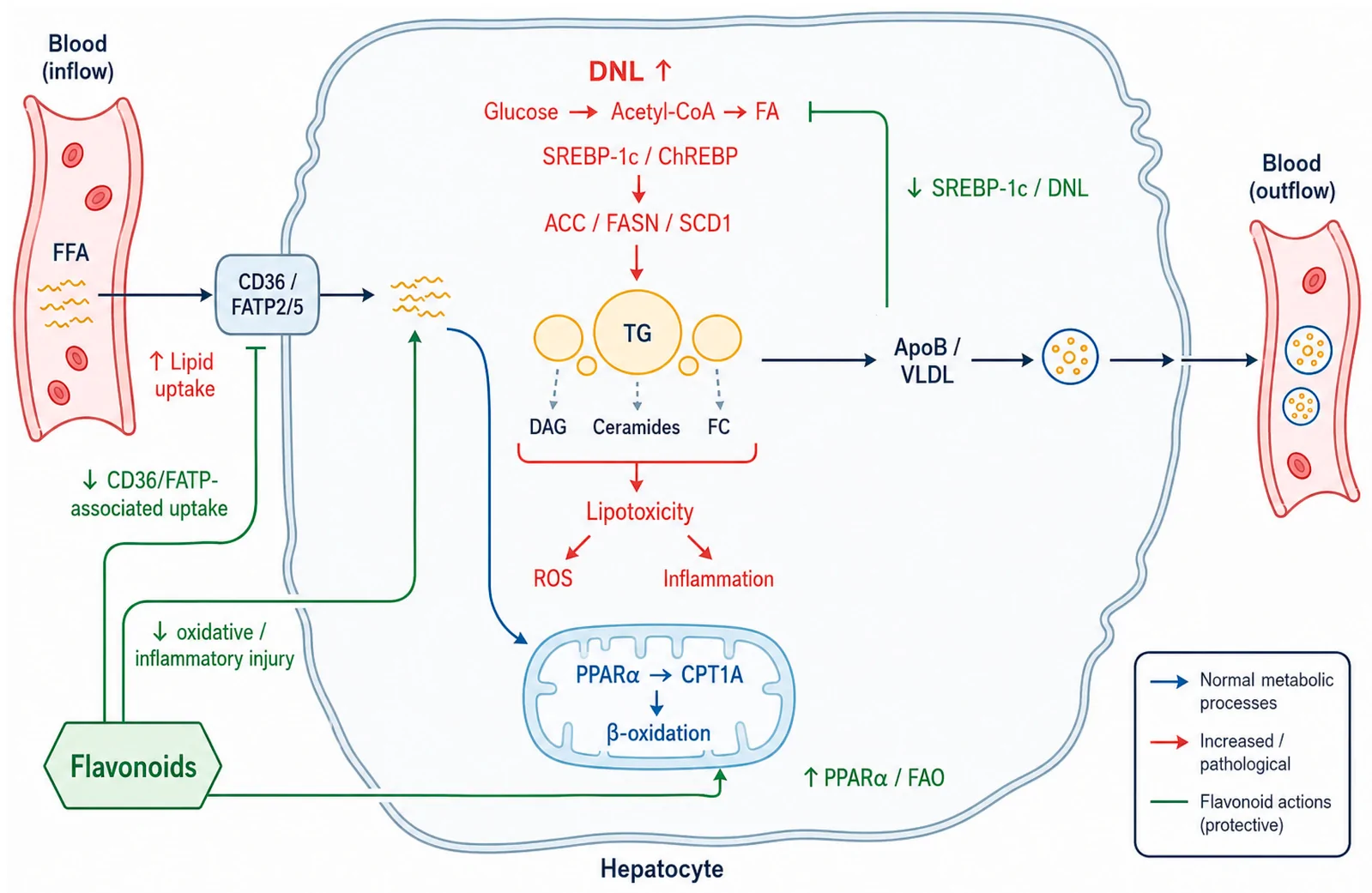
Hepatic lipid dysregulation in MASLD and the metabolic processes potentially modulated by flavonoids. Fatty-acid delivery and uptake through CD36 and FATP2/5, together with SREBP-1c- and ChREBP-driven de novo lipogenesis, expand the hepatocellular fatty-acid pool. Fatty acids are subsequently stored in lipid droplets, oxidized in mitochondria or peroxisomes, or exported in very-low-density lipoproteins. In MASLD, influx and synthesis outpace oxidation and export, leading to triglyceride accumulation and lipotoxic lipid species. Mitochondrial dysfunction, oxidative stress, insulin resistance, and inflammation reinforce this imbalance and promote progression to MASH and fibrosis. Experimental studies suggest that flavonoids may reduce lipid uptake and synthesis, enhance PPARα/CPT1A-associated oxidation, and attenuate oxidative and inflammatory stress. Arrows denote activation or metabolic flux; blunt-ended lines denote inhibition. Abbreviations: DAG, diacylglycerol; FC, free cholesterol; FFA, free fatty acid.

**Figure 2:**
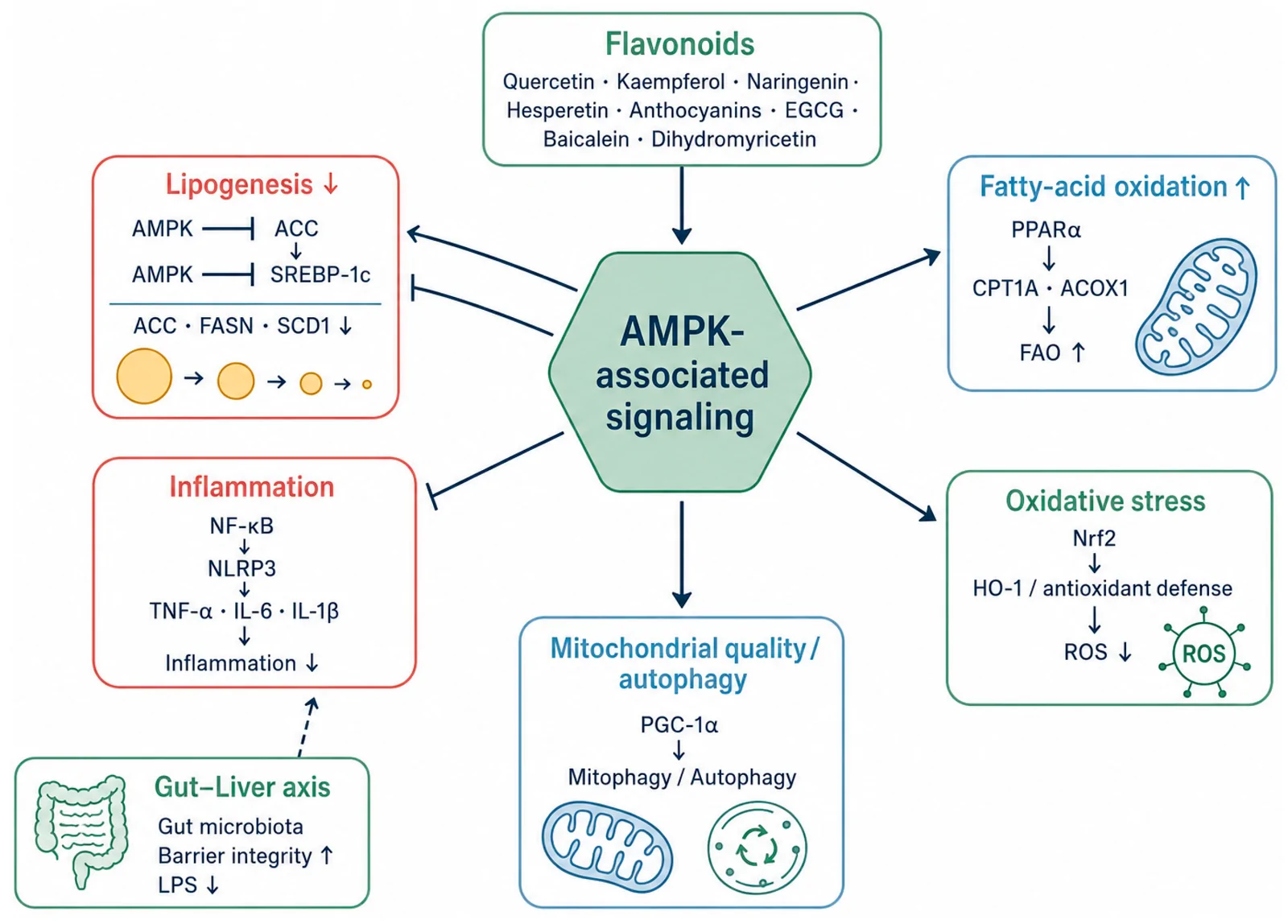
AMPK-centered network through which flavonoids may influence hepatic lipid metabolism and inflammatory stress in MASLD. AMPK is used as an integrative node, not as a universal direct target. Experimental findings connect representative flavonoids with ACC and SREBP-1c inhibition, PPARα/CPT1A/ACOX1-associated fatty-acid oxidation, mitochondrial quality control and autophagy, Nrf2-dependent antioxidant defense, and NF-κB- and NLRP3-associated inflammatory signaling. Changes in gut microbiota and intestinal barrier function may reduce portal pro-inflammatory signals, while microbial biotransformation alters flavonoid metabolite identity and exposure. MicroRNA and other post-transcriptional mechanisms represent less established regulatory layers. Solid arrows denote activation or promotion, blunt-ended lines denote inhibition, and dashed arrows denote indirect, multistep, or incompletely established relationships.

**Figure 3:**
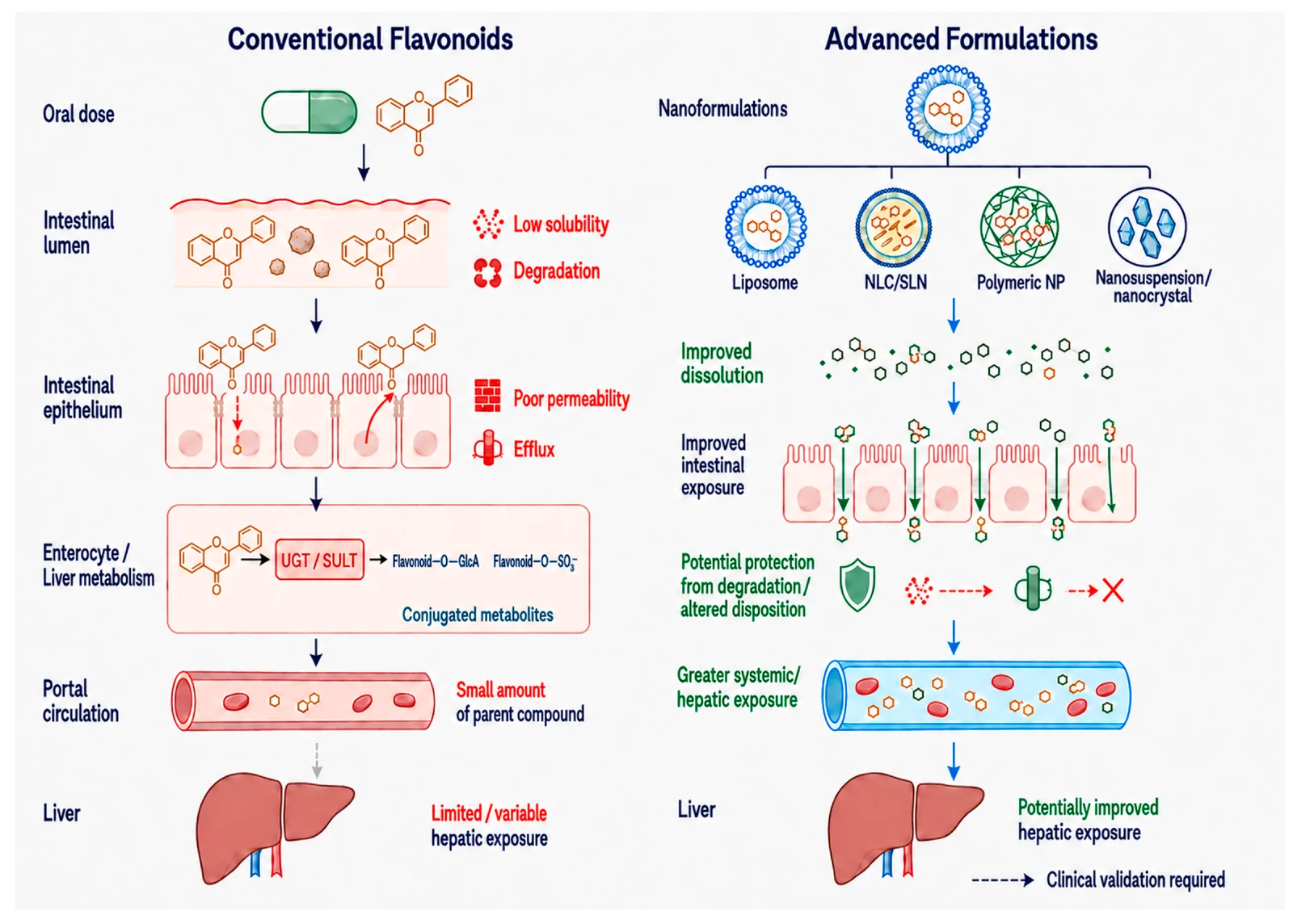
Biopharmaceutic barriers and delivery strategies for flavonoid translation in MASLD. Conventional oral products may undergo incomplete dissolution, gastrointestinal degradation, limited intestinal permeation, transporter-mediated efflux, and extensive intestinal or hepatic conjugation. The administered dose therefore does not translate directly into systemic or intrahepatic exposure to the parent compound or active metabolites. Liposomes, nanostructured lipid carriers, solid lipid nanoparticles, polymeric nanoparticles, nanosuspensions, and nanocrystals have been investigated to improve dissolution, stability, intestinal transport, pharmacokinetics, or liver distribution. Most evidence remains preclinical, and greater plasma exposure does not by itself demonstrate intrahepatic target engagement or therapeutic benefit. Improved hepatic exposure and disease modification should therefore be treated as development objectives requiring pharmacokinetic, safety, manufacturing, and clinical validation. In the schematic, solid blue arrows indicate the principal delivery or transport sequence; green arrows and labels indicate formulation-enabled improvements; red symbols and dashed red arrows denote barriers, degradation, efflux, or blocked transport; and the grey dashed arrow indicates that clinical validation remains required.

Figure 1, Figure 2 and Figure 3 and the Graphical Abstract were generated with the assistance of OpenAI’s GPT-5.6-Sol model (OpenAI, https://openai.com/, accessed on 23 July 2026) using text prompts developed by the authors. The authors determined the scientific content, reviewed and verified all pathways, molecular targets, labels, and relationships against the literature cited in this manuscript, and revised the outputs where necessary. These graphics are conceptual illustrations and do not represent experimental, clinical, microscopy, or other primary research data. No third-party figures or copyrighted images were used.

## 3. Hepatic Lipid Dysregulation as the Metabolic Core of MASLD

### 3.1. Excess Fatty-Acid Influx and Altered Hepatic Lipid Trafficking

Hepatocytes in MASLD receive an excessive fatty-acid load because insulin-resistant adipose tissue fails to suppress lipolysis fully, while dietary and intrahepatic sources add to the circulating supply. Tracer studies indicate that adipose-derived fatty acids make a major contribution to hepatic triglyceride synthesis [16]. CD36, FATP2/FATP5, and FABP1 participate in uptake and intracellular trafficking, although their relative roles vary with nutritional state, disease stage, and experimental model [28,29,30,31,32,33,34].

Rodent studies link increased hepatic CD36 expression to steatosis and dyslipidemia, whereas hepatocyte-specific CD36 disruption reduces lipid accumulation and improves insulin sensitivity [29,30,31]. Deletion or silencing of FATP5 likewise lowers hepatic triglyceride content in experimental models [32,33]. Fatty-acid import is therefore a modifiable component of the disease network, but broad transporter inhibition cannot yet be assumed to be safe or effective in humans because these proteins also support normal lipid handling.

### 3.2. De Novo Lipogenesis and Transcriptional Control by SREBP-1c and ChREBP

Increased fatty-acid influx is a major source of liver fat, but de novo lipogenesis (DNL) is also disproportionately elevated in MASLD. DNL converts carbohydrate-derived acetyl-CoA into fatty acids that are subsequently esterified into triglycerides. Under chronic overnutrition and hyperinsulinemia, this normally adaptive pathway becomes persistently active. Hepatic lipogenesis is therefore not a passive overflow route for glucose, but a regulated metabolic program with consequences for both hepatic and systemic disease [35].

SREBP-1c is the principal transcriptional driver of DNL and controls enzymes including acetyl-CoA carboxylase (ACC), fatty acid synthase (FASN), and stearoyl-CoA desaturase-1 (SCD1). Its role in the hepatic response to feeding, insulin, and liver X receptor signaling is well established [36,37]. Carbohydrate response element-binding protein (ChREBP) provides a parallel glucose-responsive input and cooperates with SREBP-1c to couple carbohydrate excess to lipid synthesis [38]. In MASLD, selective preservation of insulin′s lipogenic signaling, despite impaired control of glucose metabolism, sustains SREBP-1c activity and continued expression of ACC, FASN, and SCD1.

Human NAFLD transcriptomic studies also show dysregulation of genes involved in lipogenesis and fatty-acid handling, indicating that the pathway is not merely an artifact of animal models. DNL thus provides a direct mechanistic link between MASLD biology and a commonly reported action of flavonoids: suppression of SREBP-1c and its downstream enzyme program. This relationship is summarized in Figure 1 and Figure 2.

### 3.3. Impaired Fatty-Acid Oxidation and Mitochondrial Dysfunction

Fatty-acid oxidation normally counterbalances hepatic lipid influx and synthesis. Long-chain fatty acids enter mitochondria through carnitine palmitoyltransferase 1A (CPT1A), where β-oxidation generates acetyl-CoA and reducing equivalents. Peroxisomes handle very-long-chain fatty acids, and PPARα coordinates much of this catabolic gene network [39,40,41]. Early in MASLD, the oxidative machinery may compensate for excess substrate. When overload persists, however, fatty-acid oxidation becomes incomplete, reactive oxygen species rise, and metabolic efficiency progressively deteriorates.

The progression from steatosis to steatohepatitis is closely associated with mitochondrial failure. Studies have described defects in respiratory-chain function and membrane composition, together with excess oxidative stress, lower ATP generation, and impaired mitophagy [42,43]. Under these conditions, fatty acids are handled less safely and lipotoxic intermediates accumulate, disrupting insulin signaling and inflammatory homeostasis [14]. Inflammation and endoplasmic-reticulum stress may compound the defect by suppressing PPARα signaling and further limiting oxidative capacity.

In Figure 1, the impaired oxidative branch is represented by reduced PPARα/CPT1A-dependent fatty-acid oxidation. Figure 2 places the same defect within the broader modulation of AMPK and PPARα. Evaluating flavonoids in MASLD therefore requires evidence on mitochondrial integrity and oxidative flux in addition to evidence of reduced lipid synthesis.

### 3.4. Lipid-Droplet Turnover, VLDL Secretion, and the Paradox of Triglyceride Storage

Although triglyceride-rich droplets define hepatic steatosis histologically, triglycerides themselves are not always the most damaging lipids. In some experimental settings, their synthesis diverts free fatty acids into storage and thereby lessens lipotoxicity [17]. This temporary buffering role may help explain why simple steatosis does not inevitably progress. Disease risk appears to reflect where and in what form lipids are stored, not merely the total amount of liver fat; ceramides, diacylglycerols, and free cholesterol are of particular concern.

At the same time, hepatocytes export triglycerides in VLDL particles, whose assembly requires apolipoprotein B (apoB) and microsomal triglyceride transfer protein (MTP). VLDL secretion commonly increases in MASLD, yet it does not keep pace with substrate delivery and DNL, allowing hepatic triglycerides to continue accumulating. This mismatch also promotes systemic dyslipidemia and cardiovascular risk, connecting hepatic lipid traffic with clinically relevant extrahepatic disease [6].

### 3.5. Lipotoxicity, Insulin Resistance, Inflammation, and Disease Progression

The “multiple-hit” framework remains useful for describing progression from steatosis to MASH and fibrosis. Lipid excess creates a susceptible hepatic environment in which oxidative stress, inflammatory cytokines, gut-derived endotoxin, mitochondrial injury, endoplasmic-reticulum stress, adipokine imbalance, and genetic susceptibility act in parallel [9]. Disease severity is therefore shaped less by lipid quantity alone than by the composition of the lipid pool and the maladaptive signals generated by it.

Diacylglycerols and ceramides activate kinase pathways that impair insulin-receptor signaling, worsening hepatic insulin resistance and sustaining both dysregulated gluconeogenesis and lipogenesis [14,18]. Mitochondrial damage and oxidative stress amplify this loop. NF-κB drives cytokine production, whereas NLRP3 inflammasome signaling promotes IL-1β and IL-18 maturation. Persistent inflammatory and stress signaling eventually activates hepatic stellate cells, extracellular-matrix deposition, fibrosis, cirrhosis, and hepatocellular carcinoma [5,7,8].

Figure 1 integrates the principal disturbances in hepatic lipid handling with the metabolic processes reported to respond to flavonoids. The resulting network provides a biological rationale for multi-node modulation, but it should not be mistaken for evidence of therapeutic efficacy.

## 4. Flavonoid Chemistry and Biopharmaceutic Determinants

All flavonoids share a C6–C3–C6 scaffold, but variation in oxidation, hydroxylation, methoxylation, glycosylation, and B-ring position produces markedly different biopharmaceutic behavior. These features influence ionization, crystal packing, aqueous solubility, membrane partitioning, chemical stability, protein binding, and susceptibility to intestinal and hepatic metabolism [25,26]. Findings for a particular aglycone should not be assumed to apply to its glycosides, conjugated metabolites, or members of other flavonoid subclasses.

Quercetin and other hydroxyl-rich aglycones tend to dissolve poorly in water and are rapidly conjugated. Nobiletin and related methoxylated compounds are more lipophilic, but they face a different combination of dissolution and metabolic constraints. Glycosylation, in turn, alters intestinal uptake and susceptibility to microbial cleavage. A formulation study is interpretable only if it reports the administered chemical species, solid-state form, and excipients and identifies the analytes measured in plasma and liver; “total flavonoids” should not be treated as a single drug substance.

Gut metabolism further complicates this picture. Microorganisms can cleave flavonoid glycosides and conjugates to release aglycones or smaller phenolic products; flavonoids, in turn, can reshape community composition and barrier function. Because this exchange can alter both exposure and response, causal interpretation requires measurements resolved at the metabolite level and evidence from interventions.

## 5. Molecular Networks Linking Flavonoids to Hepatic Lipid Metabolism

### 5.1. AMPK as a Recurring Hub in Metabolic Regulation

Across experimental studies of hepatic energy metabolism, AMP-activated protein kinase (AMPK) appears repeatedly as a central regulator. Upstream activation through LKB1 or CaMKKβ restrains anabolic metabolism and affects lipogenesis, fatty-acid oxidation, mitochondrial quality control, inflammation, and autophagy [44,45]. Figure 2 is organized around AMPK to show how these processes intersect; this layout should not be read as evidence that every flavonoid binds AMPK or acts chiefly through it.

Activated AMPK phosphorylates and inhibits ACC, lowering malonyl-CoA. The immediate consequences are reduced fatty-acid synthesis and relief of CPT1A inhibition, which facilitates mitochondrial fatty-acid entry and β-oxidation. AMPK also restrains SREBP-1c and the expression of ACC, FASN, and SCD1 while supporting PPARα-dependent oxidative programs [45]. Crosstalk with Nrf2, mTOR, and autophagy pathways extends its influence beyond lipid synthesis alone.

Several flavonoid classes converge on this signaling axis in experimental systems. Quercetin activates AMPK and promotes AMPK-associated hepatic mitophagy, accompanied by less steatosis, lower expression of lipogenic proteins, and improved mitochondrial quality control [46]. Its anti-obesity and lipid-lowering actions have also been linked to AMPK signaling [47]. Baicalein activates AMPK and reduces lipid accumulation in both oleic-acid-treated HepG2 cells and high-fat-diet-fed mice [48], while anthocyanins increase AMPK phosphorylation and lower hepatocellular and hepatic triglyceride accumulation [49].

Taken together, the data identify AMPK-associated signaling as a plausible point of convergence rather than a universal primary target. Direct binding, intracellular free-compound exposure, and pathway dependence have been established in only a subset of the compounds and models examined.

### 5.2. Suppression of SREBP-1c and Lipogenic Enzymes

Suppression of SREBP-1c-dependent lipogenesis is among the more consistent responses to flavonoid exposure in MASLD models. Because SREBP-1c coordinates ACC, FASN, and SCD1 expression, its inhibition reduces both fatty-acid synthesis and monounsaturated fatty-acid production. AMPK-dependent regulation may contribute, although some flavonoids also appear to affect SREBP transcription or proteolytic maturation more directly.

Quercetin lowers hepatic SREBP-1c, ACC, and FASN expression in parallel with improved serum lipids and steatosis [46,50]. Kaempferol and kaempferide also reduce lipid accumulation and oxidative stress in hepatocyte models, with suppression of lipogenic enzymes contributing to the response [51]. Evidence from other structural classes points in the same direction: naringin couples SREBP-1c downregulation to AMPK activation, hesperetin links improved lipid homeostasis to PI3K/AKT–Nrf2 signaling [52], and genistein interferes with SREBP activation by inhibiting site-1 protease [53].

These observations place SREBP-1c and its downstream enzymes at the intersection of the disease pathway shown in Figure 1 and the flavonoid-responsive network summarized in Figure 2.

### 5.3. Enhancement of PPARα-Dependent Fatty-Acid Oxidation

PPARα coordinates genes involved in mitochondrial and peroxisomal fatty-acid oxidation, ketogenesis, and lipid catabolism. Restoring this program is relevant to MASLD because oxidative disposal becomes inadequate as disease progresses. Experimental data suggest that flavonoids may influence this pathway through several direct and indirect routes.

Naringenin is one of the better-characterized examples. In human and rat hepatic systems, it alters PPARα-, PPARγ-, and LXRα-associated signaling, with increased fatty-acid oxidation and reduced lipid and cholesterol synthesis [54]. In insulin-resistant LDLR-null mice, naringenin also reduced apoB overproduction, hyperinsulinemia, and dyslipidemia [55]. Naringin, its glycosylated precursor, improves insulin resistance and steatosis in part through PPAR-related pathways [56]. Related evidence links kaempferol to SIRT1/AMPK signaling and dihydromyricetin to AMPK/PGC-1α- and PPARα-dependent autophagy [57,58].

Increased PPARα/CPT1A/ACOX1-associated signaling could shift hepatic lipid handling toward oxidation, as depicted in Figure 1 and Figure 2. Demonstrating the same effect in humans will require metabolic-flux measurements rather than expression data alone.

### 5.4. Nrf2-Dependent Antioxidant Defense and Metabolic Crosstalk

Oxidative stress contributes to the progression from steatosis to steatohepatitis [59]. Nrf2 governs an adaptive cytoprotective program that includes heme oxygenase-1 (HO-1), NAD(P)H quinone oxidoreductase 1, glutathione-related enzymes, and superoxide dismutases. Depending on the compound and model, flavonoid-associated Nrf2 activation has been linked to the Keap1 sensor system or to upstream AMPK and PI3K/AKT signaling.

Quercetin enhances Nrf2/HO-1-associated defenses in experimental fatty liver, and hesperetin reduces oxidative and inflammatory stress through the PI3K/AKT–Nrf2–ARE pathway in cell and animal models [52]. EGCG similarly attenuates oxidative stress alongside inflammation and fibrosis [60]. These effects are unlikely to reflect radical scavenging alone: preserving mitochondrial function and limiting oxidative damage can also support fatty-acid oxidation and reduce inflammatory amplification. Figure 2 therefore presents Nrf2 as part of an interconnected metabolic and stress-response network.

### 5.5. NF-κB Inhibition and Inflammatory Amplification

Inflammatory signaling becomes increasingly important as steatosis progresses to MASH. NF-κB controls TNF-α, IL-6, MCP-1, and other mediators involved in Kupffer-cell activation, macrophage recruitment, hepatocyte stress, and stellate-cell activation. Because lipotoxicity, reactive oxygen species, and gut-derived lipopolysaccharide all converge on NF-κB, the pathway connects metabolic overload to inflammatory liver injury.

Attenuation of NF-κB signaling has been reported for several flavonoids. In high-fat-diet models, quercetin reduces IκBα and NF-κB p65 phosphorylation in parallel with improved lipid metabolism [46,61]. EGCG also limits NF-κB-associated injury [60], whereas hesperetin couples anti-inflammatory activity to improved redox control [52]. Because inflammatory cytokines suppress PPARα and exacerbate insulin resistance, dampening NF-κB may indirectly restore oxidative metabolism as well as reduce cytokine production.

### 5.6. NLRP3 Inflammasome Modulation

The NLRP3 inflammasome links lipotoxic and oxidative danger signals to caspase-1 activation, IL-1β and IL-18 maturation, and pyroptotic injury [62,63]. Flavonoid-mediated NLRP3 suppression is well described in broader inflammatory settings, but MASLD-specific evidence is less developed than that for AMPK or lipogenic pathways. NLRP3 is therefore best regarded as an emerging flavonoid-responsive target in MASLD.

### 5.7. Autophagy, Lipophagy, and Mitophagy

Autophagy maintains cellular quality and recycles substrates through processes that include lipid-droplet degradation (lipophagy) and removal of damaged mitochondria (mitophagy). Impaired autophagy in MASLD contributes to lipid retention, oxidative and endoplasmic-reticulum stress, and inflammatory signaling [64]. Restoring autophagic flux could therefore improve metabolism as well as cellular resilience.

AMPK promotes autophagy through mTOR inhibition and ULK1-related signaling, linking energy sensing to organelle quality control. Quercetin-induced AMPK-associated mitophagy provides a clear example: improved mitochondrial turnover accompanies reduced steatotic injury [46]. Dihydromyricetin has likewise been linked to AMPK/PGC-1α- and PPARα-dependent autophagy [58]. These studies justify treating autophagy as a distinct component of the network in Figure 2, while leaving its quantitative contribution to efficacy unresolved.

### 5.8. Gut–Liver Axis and Microbiota-Dependent Mechanisms

The gut–liver axis contributes to MASLD through several linked disturbances. Dysbiosis, loss of barrier integrity, altered bile-acid signaling, and the portal influx of microbial products—including lipopolysaccharide—can aggravate hepatic inflammation, insulin resistance, and disordered lipid metabolism. The relationship with flavonoids is bidirectional: intestinal microbes transform the compounds, while flavonoid exposure can reshape microbial communities and the metabolites they produce.

Quercetin provides one example of this two-way interaction. In high-fat-diet models of NAFLD, its protective activity has partly been linked to correction of dysbiosis and attenuation of gut–liver signaling [61]. Observational studies similarly associate flavonoid-rich diets with a lower prevalence of MASLD and suggest that the microbiome may mediate part of this association. In natural-product models, microbial shifts often occur alongside tighter barrier function, reduced endotoxemia, and less hepatic lipid deposition. These patterns are plausible, but only metabolite-resolved intervention studies can separate a causal effect from correlation.

### 5.9. Epigenetic and Post-Transcriptional Regulation

Some reported flavonoid actions fall outside the conventional AMPK–SREBP–PPAR framework, including effects on post-transcriptional and epigenetic control. Citrus-peel flavonoids, for example, improve lipid metabolism in HepG2 cells while reducing miR-33 and miR-122 expression [65]. Because both microRNAs regulate hepatic sterol and fatty-acid homeostasis, this observation suggests an additional regulatory layer, although the evidence remains preliminary. Figure 2 integrates these emerging mechanisms with the better-established metabolic, redox, inflammatory, autophagic, and gut–liver pathways.

## 6. Representative Flavonoids and the Strength of Supporting Evidence

### 6.1. Quercetin

Quercetin is the most extensively studied flavonoid in MASLD models. Preclinical reports describe lower lipid accumulation together with changes in AMPK, SREBP-1c, PPARα, mitochondrial quality control, Nrf2, NF-κB, cholesterol handling, and the gut microbiota [46,47,50,61]. In a crossover trial, 500 mg/day for 12 weeks produced a modest reduction in magnetic resonance imaging–proton-density fat fraction (MRI-PDFF); part of the change tracked with weight loss, and histology was not assessed [66]. Quercetin therefore has mechanistic breadth and an early clinical signal, but no demonstrated disease-modifying effect.

### 6.2. Kaempferol

Evidence for kaempferol is largely confined to cell and rodent studies. In db/db mice and oleic-acid-treated HepG2 cells, it reduced lipid accumulation through a SIRT1/AMPK-dependent mechanism supported by siRNA experiments [57]. Kaempferol and kaempferide also lowered lipid accumulation and oxidative stress in hepatocyte models [51]. Human pharmacokinetic data and MASLD intervention studies remain inadequate.

### 6.3. Naringenin and Naringin

Naringenin and its glycoside precursor naringin are citrus flavanones with a substantial preclinical metabolic literature. Naringenin modulates PPARα-, PPARγ-, and LXRα-associated pathways, increasing fatty-acid oxidation while reducing fatty-acid and cholesterol synthesis [54]. It also lowers apoB overproduction, hyperinsulinemia, and dyslipidemia in insulin-resistant models [55]. Naringin improves insulin resistance and steatosis in experimental systems, with reported AMPK activation, SREBP-1c suppression, and PPAR-related effects [56,67]. Citrus-peel extracts containing related flavonoids show similar metabolic signals [68], but extract-level findings cannot define the contribution of an individual compound. Overall, the strongest mechanistic case concerns the balance between DNL and fatty-acid oxidation, with additional effects on inflammation and insulin sensitivity.

### 6.4. Hesperetin and Hesperidin

Hesperetin and hesperidin have also been studied for hepatometabolic effects. In steatotic HepG2 cells and high-fat-diet models, hesperetin improves oxidative and inflammatory markers through PI3K/AKT–Nrf2–ARE signaling alongside changes in lipid homeostasis [52]. Hesperidin and related citrus flavonoids show hypoglycemic, hypolipidemic, and anti-adipogenic activity in other metabolic models [69,70]. The MASLD-specific evidence base is smaller than that for quercetin or naringenin, so the apparent class effect of citrus flavonoids remains provisional.

### 6.5. Anthocyanins

Anthocyanins from berries, purple sweet potato, and other pigmented foods have shown anti-steatotic activity mainly in preclinical models. Purple-sweet-potato anthocyanins activate AMPK and reduce lipid accumulation in HepG2 cells and obese mice [49]. Cyanidin-3-O-β-glucoside and related compounds also inhibit hepatic mtGPAT1 activation and fatty-acid synthesis under high-glucose conditions [71]. The available data therefore implicate both de novo lipogenesis and triglyceride assembly, although the relevance of these mechanisms to human exposure is not yet clear.

### 6.6. Epigallocatechin Gallate

Epigallocatechin gallate (EGCG), the principal green-tea catechin, has been investigated for anti-steatotic, antioxidant, anti-inflammatory, and anti-fibrotic activity. In NAFLD models, it reduces oxidative and inflammatory injury and attenuates fibrosis through pathways that include TGF/SMAD, PI3K/Akt/FoxO1, and NF-κB signaling [60]. Protection against steatosis and liver injury has also been reported in earlier obesity models [72]. Thus, the experimental literature places EGCG in both metabolic and fibrotic pathways, although poor bioavailability and safety concerns at higher doses remain substantial barriers to clinical translation.

### 6.7. Isoflavones and Related Compounds

Isoflavones broaden the range of mechanisms represented in this review. Genistein suppresses SREBP-1-regulated transcription by inhibiting site-1 protease and thereby interfering with SREBP activation [53]. In rodents, soy isoflavone or soy-protein interventions reduce hepatic lipid-droplet accumulation and alter lipid-metabolic gene expression [73]. Puerarin acts through a different route, attenuating steatosis through G-protein-coupled estrogen receptor-mediated calcium and SIRT1 signaling [74]. These observations show that relevant flavonoid actions are not restricted to AMPK-centered signaling.

### 6.8. Nobiletin and Other Polymethoxylated Flavonoids

Nobiletin is a polymethoxylated citrus flavonoid that improves obesity and insulin resistance in high-fat-diet-fed mice and downregulates ANGPTL3 in hepatic-cell and zebrafish models [75]. The findings are consistent with effects on triglyceride-rich lipoprotein metabolism and systemic lipid distribution. Its distinct methoxylated structure may also produce a different pharmacokinetic profile, an issue that deserves direct comparison rather than assumption.

### 6.9. Baicalein, Dihydromyricetin, Luteolin, Apigenin, Fisetin, and Emerging Candidates

Baicalein activates AMPK and reduces hepatic lipid accumulation in cell and animal models [48]. Dihydromyricetin improves steatosis and insulin resistance through AMPK/PGC-1α- and PPARα-dependent autophagy in experimental models [58]. In a double-blind trial of 60 adults with NAFLD, dihydromyricetin (two 150 mg capsules twice daily; 600 mg/day) for three months improved several liver-enzyme, glucose, lipid, insulin-resistance, and inflammatory biomarkers, but the study did not assess liver fat with validated imaging or histological endpoints [76]. This combination of mechanistic and early clinical evidence remains insufficient to establish disease modification.

Luteolin, apigenin, and fisetin extend coverage to additional flavone and flavonol candidates. In db/db mice, luteolin suppressed LXR–SREBP-1c signaling and hepatic de novo lipogenesis [77]. In Ldlr−/− mice, apigenin reduced hepatic lipid accumulation and atherosclerosis while inhibiting NLRP3/NF-κBsignaling in hepatocytes [78], illustrating potentially linked hepatic and extrahepatic effects. Fisetin improved steatosis, glucose and lipid profiles, antioxidant signaling, and gluconeogenic regulation in high-fat-diet-fed mice [79].These findings broaden the compound spectrum, but they remain preclinical and do not establish class-wide efficacy. The comparative evidence is summarized in Table 1.

### 6.10. Scope Boundary

Curcumin and berberine were excluded from the comparative efficacy synthesis because neither is a flavonoid. They remain useful external comparators for natural-product formulation, but combining them with flavonoids would weaken chemical-class-specific interpretation. Sources addressing MASLD biomarkers [88], vitamin A metabolism [89], hypoxia, asprosin, or broader cardiometabolic disease [90,91,92], botanical mixtures [93,94], antioxidant synergy [95], antiviral activity [96], and plant physiology [97] were retained for context or scope transparency and were not treated as direct evidence of compound-level efficacy in MASLD.

## 7. Clinical Evidence: Signals, Limitations, and Evidentiary Gaps

Human evidence remains sparse, although several intervention studies now provide early signals. Quercetin at 500 mg/day for 12 weeks produced a modest MRI-PDFF reduction in a randomized crossover trial, with little change in most secondary outcomes [66]. Separate 12-week studies of hesperidin (1 g/day) and purified anthocyanins (320 mg/day) reported improvements in selected liver, inflammatory, or metabolic markers [80,81]. Small trials of catechin-rich green tea or green-tea extract also yielded biochemical or imaging signals [82,83], and a 2024 randomized translational study implicated dipeptidyl peptidase 4 (DPP4) in the response to EGCG [84]. Soy-isoflavone studies reported changes in lipids, controlled attenuation parameter, or steatosis grade, but enrolled only 50 participants and lasted 12 weeks [87]. A three-month dihydromyricetin trial reported improvements in selected biochemical and inflammatory markers but did not use validated liver-fat or histological endpoints [76].

Interpretation is limited by substantial heterogeneity in flavonoid composition, dose, background lifestyle advice, diagnostic criteria, and endpoint selection. Most trials were short, single-center studies without power to assess fibrosis; several relied on liver enzymes, ultrasonography, or other surrogate measures. None demonstrates MASH resolution, fibrosis regression, prevention of clinical events, or equivalence to approved treatment. Concordant movement in a few biomarkers should therefore not be equated with established clinical efficacy.

An updated systematic review and meta-analysis of 25 randomized controlled trials involving 1689 participants found improvements in several liver-enzyme and metabolic measures, but no significant effects on hepatic steatosis or fibrosis outcomes; intervention periods ranged from 4 to 24 weeks, with 12 weeks the most frequent duration [98]. These pooled findings reinforce the distinction between short-term biomarker changes and clinically meaningful modification of MASH or fibrosis.

The discrepancy between extensive mechanistic activity and modest clinical efficacy is unlikely to have a single explanation. Limited systemic or hepatic exposure is one plausible contributor, but preclinical concentrations may also exceed clinically attainable free-compound levels; circulating conjugates may not reproduce the actions of parent aglycones; animal and nutrient-deficient models may not capture human disease heterogeneity; and short trials may use endpoints that are insensitive to fibrosis remodeling. Weight change, background lifestyle care, small samples, incomplete adherence assessment, heterogeneous products, and selective pathway reporting add further uncertainty. Figure 3 therefore presents improved exposure as a testable formulation hypothesis rather than a sufficient explanation. Future trials should pair validated liver outcomes with metabolite-resolved pharmacokinetics, target-engagement measures, and exposure–response analysis.

Duration is a specific evidentiary limitation. Trials lasting 4–24 weeks can detect enzyme or metabolic changes but are generally too short to establish sustained liver-fat reduction, MASH resolution, or fibrosis regression [98]. Proof-of-concept studies should include serial quantitative imaging and follow-up beyond the initial exposure period, whereas trials intended to assess histological disease modification should use durations and endpoint schedules comparable with contemporary MASLD drug-development programs, together with longer safety extensions when chronic supplementation is proposed [19,20]. Selected randomized human studies are summarized in Table 2.

## 8. Translational Barriers: From Nominal Dose to Hepatic Exposure

### 8.1. Low Oral Bioavailability

Poor aqueous solubility, limited intestinal permeability, rapid glucuronidation or sulfation, and transporter-mediated efflux restrict oral exposure for many flavonoids. Consequently, free-aglycone concentrations used in vitro may exceed those attainable in patients. Naringenin illustrates these limitations and has consequently received considerable pharmacokinetic and formulation attention [99]. Bioavailability estimates, however, are meaningful only when the measured analytes are chemically defined; nominal dose alone is not an adequate surrogate.

These constraints are summarized in the left panel of Figure 3. With conventional oral administration, incomplete dissolution, presystemic metabolism, and transporter-mediated efflux may each limit exposure. These factors may help explain why persuasive mechanistic data have not consistently translated into strong clinical effects.

### 8.2. Hepatic Exposure and Metabolite Identity

For a liver-directed treatment, total plasma concentration may be a poor proxy for pharmacologically relevant exposure. The amount reaching hepatocytes is shaped by portal delivery, metabolic conversion, protein binding, cellular uptake, intracellular deconjugation, and, for some flavonoids, microbial transformation. This matters because conjugated metabolites often circulate at concentrations above those of the parent aglycone yet can differ markedly in cellular activity. Translational studies should therefore characterize hepatic pharmacokinetics and metabolite profiles and demonstrate target engagement, rather than infer efficacy from oral dose or total plasma exposure.

### 8.3. Safety and Interaction Considerations

Experience with flavonoids consumed as foods does not establish the safety of concentrated extracts or nanocarrier formulations. EFSA reported a liver-safety concern for green-tea supplements supplying about 800 mg/day or more of EGCG [85]. In a large randomized intervention, long-term intake of 843 mg/day EGCG produced liver-enzyme responses that varied with genotype [86]. Quercetin and its metabolites interact with drug-metabolizing enzymes and transporters in vitro, while human probe-drug studies show that high supplemental doses can alter CYP-mediated pharmacokinetics [100]. Additional demerits requiring explicit evaluation include gastrointestinal intolerance, product-to-product variability, contamination or adulteration, pro-oxidant effects at high exposure, and carrier-related immune, organ-accumulation, or excipient toxicity. Short trials are not sufficient to exclude uncommon or cumulative harms. MASLD trials should therefore prespecify product composition, dosing conditions, concomitant medications, liver and renal safety monitoring, stopping rules, and nanocarrier-specific toxicity assessment.

### 8.4. Dose, Formulation, and Exposure–Response

Nominal milligram doses cannot be compared directly across flavonoids because molecular mass, glycosylation, purity, extract composition, food matrix, and formulation all influence the delivered molar dose and active-metabolite exposure. Across the trials summarized in the updated meta-analysis, doses ranged from 32 to 1080.6 mg/day, yet heterogeneity precluded a consistent class-wide dose–response interpretation [98]. Future studies should report aglycone equivalents and full product composition, justify dose selection using human pharmacokinetic data, measure parent compounds and conjugated or microbial metabolites, and relate exposure to both target engagement and toxicity. Escalating the nominal dose without these measurements may increase adverse effects without improving hepatic exposure.

## 9. Formulation and Delivery Strategies for Flavonoid Translation

Figure 3 contrasts the liabilities of conventional oral products with the aims of advanced delivery. The relevant question is not simply whether a carrier increases total plasma exposure, but whether it improves dissolution, stability, intestinal transport, active-metabolite exposure, liver distribution, and therapeutic index in a reproducible manner. A recent *Pharmaceutics* review likewise emphasizes formulation composition, manufacturing method, physicochemical characterization, and translational limitations rather than treating nanotechnology as a generic solution [27].

Naringenin currently provides the most coherent MASLD-focused formulation evidence. In methionine- and choline-deficient (MCD)-fed mice, nanoliposomes improved oral absorption and produced effects comparable with free naringenin at one-quarter of the dose [101]. Nanostructured lipid carriers with a mean size of approximately 172 nm, 23.7% drug loading, and 99.9% encapsulation efficiency increased transport and systemic exposure and reduced hepatic lipid accumulation in cell and mouse models [102]. Nanosuspensions and polymeric nanoparticles also improved dissolution and relative oral exposure in rats, but neither approach has been tested in human MASLD [103,104].

For quercetin, glycyrrhizic-acid-stabilized nanocrystals achieved greater liver distribution than poloxamer-stabilized nanocrystals after intravenous administration, although the study did not establish efficacy in MASLD [105]. Solid-lipid naringenin nanoparticles subsequently produced a 9- to 12-fold increase in oral bioavailability in rats [106], while chitosan-encapsulated quercetin nanoparticles improved biochemical and histological features in an HFD rat model [107]. The studies support the formulation rationale, but none moves the evidence beyond the preclinical stage.

No flavonoid nanocarrier has demonstrated clinical efficacy in MASLD. Development priorities include batch reproducibility, solid-state and colloidal stability, release under biorelevant conditions, metabolite-resolved pharmacokinetics, tissue distribution, repeated-dose nanotoxicology, scalable manufacturing, and direct comparison with the free compound. Any depiction of fibrosis regression in Figure 3 should be understood as a therapeutic goal, not an observed outcome. Representative formulation studies are summarized in Table 3.

## 10. Future Directions

Mechanistic studies would be more informative if they used exposure-matched concentrations and human-relevant systems, including primary hepatocytes, organoids, and multicellular models. Parent compounds, conjugates, and microbial metabolites should be quantified, and pathway dependence should be tested rather than inferred from expression changes. Animal experiments should also distinguish reduced neutral-lipid storage from reduced lipotoxic injury and incorporate models that reproduce insulin resistance and fibrotic MASH instead of relying predominantly on nutrient-deficient diets.

Formulation development should begin with a target product profile rather than a preferred carrier. Relevant attributes include drug loading, encapsulation efficiency, particle-size distribution, release in biorelevant media, gastrointestinal stability, hepatic exposure, and safety margin. Head-to-head studies must then determine whether improved exposure produces proportional target engagement and efficacy; a higher AUC is not intrinsically beneficial.

Clinical translation will require chemically standardized products, longer follow-up, adequate power, and validated endpoints such as MRI-PDFF, elastography, fibrosis biomarkers, and, when justified, paired histology. The 4–24-week durations that dominate the current literature are inadequate for establishing sustained MASH or fibrosis benefit [98]. Stratification by fibrosis stage, diabetes, obesity, sex, genotype, microbiome features, and concomitant resmetirom or semaglutide may help identify responders. Early-phase studies should integrate population pharmacokinetics with exposure–response modeling, whereas later trials should use endpoint timing aligned with contemporary standards and include longer safety follow-up for products intended for chronic use [19,20].

Implementation studies should address batch-to-batch chemical equivalence, good-manufacturing-practice production, storage stability, scalable carrier manufacture, adherence, food and drug interactions, cost, and compatibility with standard MASLD care. Pragmatic studies and prospective registries could then test whether exposure, tolerability, and effectiveness observed under controlled conditions persist in routine care. Predefined quality specifications and pharmacovigilance are especially important for multicomponent extracts and nanocarriers.

Because MASLD is a multisystem cardiometabolic disorder, future studies should assess extrahepatic outcomes as well as liver endpoints. Preclinical findings with naringin, nobiletin, and apigenin suggest possible effects on dyslipidemia, insulin resistance, cardiovascular dysfunction, or atherosclerosis [67,75,78], but these systemic changes may reflect weight loss or broad metabolic effects rather than liver-specific target engagement. Trials should therefore prespecify blood pressure, atherogenic lipoproteins, glycemic control, renal function, body composition, and cardiovascular events or validated risk markers, while distinguishing systemic benefit from hepatic disease modification [6].

## 11. Conclusions

MASLD reflects disordered hepatic lipid flux within a broader cardiometabolic and fibro-inflammatory disease. Experimental findings link flavonoids to changes in lipid uptake and synthesis, fatty-acid oxidation, mitochondrial quality control, redox and inflammatory signaling, autophagy, and the gut–liver axis. These observations provide a set of testable mechanisms, but the dominant pathway is likely to vary with the compound, metabolite, dose, model, and stage of disease.

Human trials presently show early signals in liver fat or biochemical markers, not MASH resolution or fibrosis regression. Formulation studies, particularly those involving naringenin, demonstrate that systemic exposure and liver distribution can be increased, but whether those gains translate into clinical benefit has not been tested adequately. A defensible development path must integrate chemical standardization, exposure-matched mechanistic work, quantitative biopharmaceutics, safety assessment, scalable manufacture, and trials with contemporary liver endpoints. Until that evidence is available, flavonoids are best described as investigational, formulation-dependent candidates for MASLD rather than established therapies.

## Figures and Tables

**Table 1:** Representative flavonoids, experimental context, and level of evidence in MASLD.

Compound/Class	Models	Main Signal	Proposed Mechanisms	Evidence Level	Refs.
Quercetin	HepG2; rodent MASLD; small human crossover trial	Reduced experimental steatosis; modest MRI-PDFF reduction in humans	AMPK, SREBP-1c, mitophagy, Nrf2, NF-κB; microbiota	Preclinical evidence + early clinical signal	[46,47,50,61,66]
Kaempferol	HepG2; db/db mice	Reduced lipid accumulation and liver injury	SIRT1/AMPK dependence; reduced ACC/FASN/SREBP-1c	Preclinical	[51,57]
Naringenin/naringin	Hepatic cells; insulin-resistant and NAFLD rodents	Improved lipid handling and steatosis-related outcomes	PPARα/PPARγ/LXRα; apoB; DNL–FAO balance	Preclinical	[54,55,56,67,68]
Hesperetin/hesperidin	HepG2; HFD rodents; small RCT	Improvements in selected liver and metabolic markers	PI3K/AKT–Nrf2; inflammatory signaling	Preclinical evidence + early clinical signal	[52,69,70,80]
Anthocyanins	HepG2; obese mice; pilot RCT	Reduced lipid accumulation; improved selected injury markers	AMPK; mtGPAT1; substrate partitioning	Preclinical evidence + early clinical signal	[49,71,81]
EGCG/green-tea catechins	Rodents; small RCTs	Improvements in selected imaging or biochemical markers; dose-related safety concerns	Redox, NF-κB, TGF/SMAD, DPP4	Preclinical evidence + early clinical signal	[60,72,82,83,84,85,86]
Soy isoflavones/ puerarin	Rodents; small RCTs	Improvements in selected lipid, CAP, or steatosis outcomes	SREBP processing; GPER–Ca^2+^–SIRT1 signaling	Preclinical evidence + early clinical signal	[53,73,74,87]
Baicalein/dihydromyricetin	HepG2; rodent models; small human RCT	Reduced experimental lipid accumulation and insulin resistance; selected biochemical signals in humans	AMPK; PGC-1α; PPARα-mediated autophagy	Preclinical evidence + early clinical signal	[48,58,76]
Luteolin/apigenin/fisetin	HepG2; primary hepatocytes; genetic and diet-induced mouse models	Reduced steatosis-related outcomes; apigenin also reduced atherosclerosis in mice	LXR–SREBP-1c; NLRP3/NF-κB; GSK-3β/Nrf2/HO-1; gluconeogenic regulation	Preclinical	[77,78,79]

Evidence levels are descriptive and do not represent a formal risk-of-bias grade. Abbreviations: ACC, acetyl-CoA carboxylase; AKT, protein kinase B; AMPK, AMP-activated protein kinase; apoB, apolipoprotein B; Ca^2+^, calcium ion; CAP, controlled attenuation parameter; db/db, leptin receptor-deficient mouse model; DNL, de novo lipogenesis; DPP4, dipeptidyl peptidase-4; EGCG, epigallocatechin gallate; FAO, fatty-acid oxidation; FASN, fatty acid synthase; GPER, G protein-coupled estrogen receptor; GSK-3β, glycogen synthase kinase-3 beta; HepG2, human hepatocellular carcinoma cell line; HFD, high-fat diet; HO-1, heme oxygenase-1; LXR, liver X receptor; MASLD, metabolic dysfunction-associated steatotic liver disease; MRI-PDFF, magnetic resonance imaging–proton-density fat fraction; mtGPAT1, mitochondrial glycerol-3-phosphate acyltransferase 1; NAFLD, non-alcoholic fatty liver disease; NF-κB, nuclear factor kappa B; NLRP3, NOD-like receptor family pyrin domain-containing 3; Nrf2, nuclear factor erythroid 2-related factor 2; PGC-1α, peroxisome proliferator-activated receptor gamma coactivator 1-alpha; PI3K, phosphoinositide 3-kinase; PPAR, peroxisome proliferator-activated receptor; RCT, randomized controlled trial; SIRT1, sirtuin 1; SMAD, small mothers against decapentaplegic; SREBP, sterol regulatory element-binding protein; SREBP-1c, sterol regulatory element-binding protein 1c; TGF, transforming growth factor.

**Table 2:** Selected randomized human studies of flavonoid interventions in NAFLD/MASLD.

Intervention	Design	Exposure	Endpoints	Main Finding and Limitation	Ref.
Quercetin	Randomized double-blind crossover; n = 41	500 mg/day; 12 weeks per period	MRI-PDFF; liver tests; safety	Modest MRI-PDFF reduction; association with weight loss; no histological assessment	[66]
Hesperidin	Randomized double-blind placebo-controlled; n = 50	1 g/day; 12 weeks + lifestyle advice	Ultrasound steatosis; ALT/GGT; lipids; inflammatory markers	Improvements in selected endpoints; small, short, single-center trial	[80]
Purified anthocyanins	Randomized double-blind pilot; n = 74	320 mg/day; 12 weeks	ALT; CK-18 M30; glucose; lipids	Improvements in selected injury and glucose markers; no validated fibrosis endpoints	[81]
Catechin-rich green tea	Randomized double-blind placebo-controlled; n = 17	>1 g catechins/day in 700 mL; 12 weeks	CT liver:spleen ratio; ALT; 8-isoprostane	Imaging and biochemical signals; very small sample	[82]
Green-tea extract	Randomized double-blind placebo-controlled; n = 80	500 mg/day; 90 days	ALT/AST/ALP; ultrasound diagnosis	Reported enzyme improvement; limited extract characterization and no advanced liver endpoints	[83]
Soy isoflavones	Randomized placebo-controlled; n = 50	100 mg/day; 12 weeks	Lipids; CAP; enzymes; steatosis grade	Selected metabolic and steatosis signals; short duration and small sample	[87]
Dihydromyricetin	Randomized double-blind placebo-controlled; n = 60	600 mg/day; 12 weeks	Liver enzymes; glucose/lipids; HOMA-IR; inflammatory biomarkers	Improvement in selected biochemical and inflammatory markers; no validated imaging or histological endpoint	[76]

The studies used NAFLD terminology at publication; none established MASH resolution or fibrosis regression. Abbreviations: ALP, alkaline phosphatase; ALT, alanine aminotransferase; AST, aspartate aminotransferase; CAP, controlled attenuation parameter; CK-18, cytokeratin-18; CT, computed tomography; GGT, gamma-glutamyl transferase; HOMA-IR, homeostatic model assessment for insulin resistance; M30, caspase-cleaved cytokeratin-18 fragment; MASH, metabolic dysfunction-associated steatohepatitis; MRI-PDFF, magnetic resonance imaging–proton-density fat fraction; n, sample size; NAFLD, non-alcoholic fatty liver disease.

**Table 3:** Representative flavonoid formulation studies relevant to hepatic delivery and MASLD.

System	Key Attributes	Pharmaceutic Outcome	Disease/Model Evidence	Main Limitation	Ref.
Naringenin nanoliposome	Sustained-release liposomal formulation	Increased oral absorption; comparable effect at one-quarter free-drug dose	MCD-diet mouse NAFLD	No human PK, chronic safety, or scalability data	[101]
Naringenin NLC	171.9 nm; 23.7% loading; 99.9% encapsulation; 86.2% release	Increased transport, Cmax and AUC	Primary hepatocytes and MCD-diet mice	Nutrient-deficient model; no clinical validation	[102]
Naringenin nanosuspension	117 nm; PVP K-90 stabilized	Dissolution 91% vs. 42%; Cmax ~2-fold and AUC ~1.8-fold higher	Rat PK; no MASLD efficacy study	Limited stability and food-effect data	[103]
Naringenin polymeric NPs	PLA/PVA or zein/pectin matrices	Relative bioavailability 4.7-fold or 1.9-fold	Single-dose rat PK	No disease-specific efficacy or repeated-dose safety	[104]
Quercetin nanocrystals	~130 nm; glycyrrhizic-acid stabilization	Greater liver distribution than poloxamer control after IV dosing	Rat tissue distribution	IV route; no MASLD efficacy or human data	[105]
Naringenin SLNs	74–91 nm; 79–85% entrapment	Approximately 9–12-fold higher oral bioavailability	Rat PK and general safety	No MASLD-specific efficacy or human validation	[106]
Quercetin–chitosan NPs	~190 nm; +56.5 mV; 65% encapsulation	Improved biochemical and histological outcomes	HFD-rat MASLD model	No comparative PK, fibrosis endpoint, or human data	[107]

Abbreviations: AUC, area under the concentration–time curve; Cmax, maximum plasma concentration; HFD, high-fat diet; IV, intravenous; MASLD, metabolic dysfunction-associated steatotic liver disease; MCD, methionine- and choline-deficient; NLC, nanostructured lipid carrier; NP, nanoparticle; PK, pharmacokinetics; PLA, poly(lactic acid); PVA, poly(vinyl alcohol); PVP, polyvinylpyrrolidone; SLN, solid lipid nanoparticle.

## Data Availability

No new data were created or analyzed in this study. Data sharing is not applicable to this article.

## Abbreviations

The following abbreviations are used in this manuscript:

ACC	acetyl-CoA carboxylase
ACOX1	acyl-CoA oxidase 1
ALP	alkaline phosphatase
ALT	alanine aminotransferase
AMPK	AMP-activated protein kinase
apoB	apolipoprotein B
AST	aspartate aminotransferase
AUC	area under the concentration–time curve
CAP	controlled attenuation parameter
ChREBP	carbohydrate response element-binding protein
CK-18	cytokeratin 18
Cmax	maximum plasma concentration
CPT1A	carnitine palmitoyltransferase 1A
CT	computed tomography
DAG	diacylglycerol
DNL	de novo lipogenesis
DPP4	dipeptidyl peptidase 4
EGCG	epigallocatechin gallate
FA	fatty acid
FABP1	fatty acid-binding protein 1
FAO	fatty-acid oxidation
FASN	fatty acid synthase
FATP	fatty acid transport protein
FC	free cholesterol
FFA	free fatty acid
GGT	γ-glutamyltransferase
GPER	G-protein-coupled estrogen receptor
HCC	hepatocellular carcinoma
HFD	high-fat diet
HO-1	heme oxygenase 1
IV	intravenous
LPS	lipopolysaccharide
MASLD	metabolic dysfunction-associated steatotic liver disease
MASH	metabolic dysfunction-associated steatohepatitis
MCD	methionine- and choline-deficient
MTP	microsomal triglyceride transfer protein
MRI-PDFF	magnetic resonance imaging proton-density fat fraction
NAFLD	nonalcoholic fatty liver disease
NF-κB	nuclear factor kappa B
NLC	nanostructured lipid carrier
NLRP3	NOD-like receptor protein 3
NP	nanoparticle
Nrf2	nuclear factor erythroid 2-related factor 2
PGC-1α	peroxisome proliferator-activated receptor gamma coactivator 1-alpha
PK	pharmacokinetics
PLA	polylactic acid
PPARα	peroxisome proliferator-activated receptor α
PVA	polyvinyl alcohol
PVP	polyvinylpyrrolidone
ROS	reactive oxygen species
SCD1	stearoyl-CoA desaturase 1
SLN	solid lipid nanoparticle
SREBP-1c	sterol regulatory element-binding protein 1c
SULT	sulfotransferase
TG	triglyceride
UGT	UDP-glucuronosyltransferase
VLDL	very-low-density lipoprotein
